# Evaluating public health campaigns on health promotion, substance use prevention and physical activity: a systematic review

**DOI:** 10.1093/her/cyag008

**Published:** 2026-02-27

**Authors:** Thomas Zandonai, Giulia Scarpa, Vittoria Barbati, Alessandro Carollo, Andrea Bizzego, Gianluca Esposito, Ornella Corazza

**Affiliations:** Addiction Science Lab, Department of Psychology and Cognitive Science, University of Trento, Corso Bettini, 84, 38068 Rovereto, Trento, Italy; Pharmacology, Health, and Addiction in Exercise Laboratory (PHAE Lab), Department of Pharmacology, Paediatrics and Organic Chemistry, Miguel Hernández University of Elche, Crta. Nacional, N-332. s/n, 03550 Sant Joan, Alicante, Spain; Addiction Science Lab, Department of Psychology and Cognitive Science, University of Trento, Corso Bettini, 84, 38068 Rovereto, Trento, Italy; Addiction Science Lab, Department of Psychology and Cognitive Science, University of Trento, Corso Bettini, 84, 38068 Rovereto, Trento, Italy; Addiction Science Lab, Department of Psychology and Cognitive Science, University of Trento, Corso Bettini, 84, 38068 Rovereto, Trento, Italy; Addiction Science Lab, Department of Psychology and Cognitive Science, University of Trento, Corso Bettini, 84, 38068 Rovereto, Trento, Italy; Addiction Science Lab, Department of Psychology and Cognitive Science, University of Trento, Corso Bettini, 84, 38068 Rovereto, Trento, Italy; Addiction Science Lab, Department of Psychology and Cognitive Science, University of Trento, Corso Bettini, 84, 38068 Rovereto, Trento, Italy; School of Health, Medicine and Life Sciences, University of Hertfordshire, United Kindgom

## Abstract

Public health campaigns on substance use and physical activity aid disease prevention. This review examines how process evaluations are conducted in substance use and physical activity campaigns, focusing on methodologies, theoretical frameworks, implementation quality, fidelity, reach, and delivery. Following Preferred Reporting Items for Systematic Reviews and Meta-Analyses guidelines, a comprehensive literature search was conducted across PubMed, PsycINFO, Web of Science, Scopus, ProQuest, and Google Scholar from 1990 up to April 2025. Eligible studies included randomized controlled trials, quasi-experimental, and observational designs involving adult human participants and reporting process or implementation evaluation components. Twenty-one studies met inclusion criteria. A one-step forward citation analysis was performed using the *bibliometrix* package in R. Findings revealed that 62% of studies reported process evaluation components, with 52% employing mixed-method approaches. Commonly cited theoretical frameworks included the Transtheoretical Model, Theory of Planned Behaviour, and Social Cognitive Theory. Only 20% of studies explicitly defined process evaluation objectives, and few translated findings into actionable implementation adaptations. Bibliometric analysis indicated influence across 373 publications, primarily from the United States, Australia, and Canada. Overall, process evaluation in public health campaigns remain inconsistent. To enhance the quality and utility of such evaluations, greater theoretical integration, improved methodological transparency, and the use of standardized assessment tools are recommended.

## Introduction

Regular physical activity is widely promoted for its well-established physical and mental health benefits [1–3]. It is associated with the prevention of chronic diseases, improvements in psychological well-being, and overall enhancements in quality of life [4, 5]. However, growing evidence suggests that individuals engaged in sports, both recreational and professional, may be at increased risk of using image-and performance-enhancing substances (IPEDs), an umbrella term referring to a broad range of legal and illegal substances, such as anabolic steroids and medicinal products used without prescription, stimulants, or unregulated dietary supplements consumed to enhance physical performance or alter body image in response to social and cultural pressures [6–9]. This paradox, where a health-promoting behaviour like physical activity becomes linked with risky substance use, raises important public health concerns. Such behaviours are not confined to elite athletes, but are increasingly observed among adolescents and amateur gym-goers, frequently driven by distorted body ideals promoted on social media and widespread misinformation [10–12].

In response to the increasing concerns related to substance use, body dissatisfaction, and physical inactivity, public health campaigns have become essential preventive strategies. Through mass media, social platforms, and community-based outreach, these interventions aim to raise awareness, shift social norms, and promote healthier behaviours [13]. When effectively implemented, public health campaign can influence attitudes and influence healthier behaviours across diverse populations. Recent empirical studies support their effectiveness, particularly when utilizing multi-channel approaches. Interventions focusing on physical activity, body image, and substance use have shown significant improvements in behavioural intentions and perceived norms, particularly among adolescents and young adults [14]. Digital campaigns that incorporate tailored messaging, cultural sensitivity, and interactive elements further enhance audience engagement and self-reported behaviour change [15]. These results are reinforced by systematic reviews which indicate that well-structured, multi-component campaigns, characterized by coherent messaging and strategically targeted outreach, can achieve meaningful behavioural outcomes, particularly among younger populations [16].

However, the effectiveness of these initiatives depends not only on content quality but also on the consistency and rigour of implementation. Robust evaluation is critical in this context, offering a systematic framework to assess the design, delivery, and impact of interventions as well as elucidate the mechanisms driving observed outcomes [17]. Within public health communication, evaluation fulfils three critical roles: measuring intended effects, examining operational functionality, and guiding future planning. It also facilitates iterative improvement by identifying successful components and pinpointing areas needing adjustment [18]. Contemporary models, such as the CDC’s 2024 Program Evaluation Framework, promote an integrated approach encompassing formative, process, and outcome evaluations throughout the program lifecycle [19]. This is exemplified by the The Together Everyone Achieves More Physical Activity (TEAM-PA) randomized trial, which employed iterative process evaluation to optimize delivery strategies in real time, enhancing adherence and program effectiveness among African American women [20].

Process evaluation, in particular, plays a pivotal role in determining whether campaigns are implemented as intended. It systematically examines key dimensions such as fidelity, reach, exposure, and participant engagement, providing crucial insights into the quality and effectiveness of implementation [21]. Evidence from systematic reviews of sports and community-based health promotion initiatives underscores the importance of these factors, alongside stakeholder involvement and adaptability to local contexts, in shaping program outcomes [22]. Despite its recognized value, process evaluation is often inconsistently applied and inadequately reported in campaigns targeting substance use and physical activity. Moreover, the lack of explicit theoretical frameworks limits the ability to refine and replicate effective interventions based on empirical evidence.

This review examines how public health campaigns addressing substance use and physical activity conduct process and implementation evaluations. Specifically, we assess the methodologies, theoretical frameworks, and implementation strategies employed in these evaluations, rather than the campaigns' effectiveness or outcomes.

## Methods

A systematic review was carried out following the Preferred Reporting Items for Systematic Reviews and Meta-Analyses (PRISMA) guidelines to ensure transparency and rigour in study identification, selection, and synthesis [23]. The review protocol was pre-registered on PROSPERO (CRD420251054292).

### Literature search

A comprehensive search was conducted in PubMed, PsycINFO, Web of Science, Scopus, and grey literature sources (ProQuest and Google Scholar) from January 1990 up to April 2025. The following Boolean search strategy was used: (‘process evaluation’ OR ‘implementation evaluation’) AND (‘campaign’ OR ‘marketing’ OR ‘media’ OR ‘video’) AND (‘body image’ OR ‘body dissatisfaction’ OR ‘dysmorphia’ OR ‘body disorder’ OR ‘physical activity’ OR ‘exercise’ OR ‘supplements’ OR diuretics” OR ‘anabolic androgenic steroids’ OR ‘stimulants’ OR ‘enhancing drugs’ OR ‘psychoactive’ OR ‘substance’ OR ‘doping’).

We employed the broad Boolean “OR” operator to ensure comprehensive retrieval of campaigns addressing substance use, physical activity, or body image-behavioural domains that, while conceptually distinct, are increasingly recognized as interconnected in public health practice. This approach was particularly important for capturing campaigns targeting athletic populations, where physical activity promotion may coexist with substance misuse risks such as performance-enhancing drug use.

Beyond database searches, the reference lists of all included articles were manually screened to identify relevant studies potentially omitted due to alternative terminology or indexing. This backward citation tracking was supplemented by targeted searches in ProQuest Dissertations & Theses and Google Scholar to identify grey literature and unpublished studies meeting predefined inclusion criteria, thereby reducing publication bias and broadening the evidence base. To ensure cross-database compatibility, we used free-text keywords rather than database-specific controlled vocabularies (*e.g.* Medical Subject Headings: MeSH), as our search encompassed multiple platforms with differing indexing systems (PubMed, PsycINFO, Web of Science, Scopus, ProQuest).

### Inclusion and exclusion criteria

Studies were deemed eligible for inclusion if they focused on the process or implementation evaluation of public health campaigns addressing either substance use or physical activity. Eligible studies comprised original research using randomized controlled trials, quasi-experimental designs, or observational methods (cross-sectional, cohort, case–control). Peer-reviewed and unpublished full-text works with sufficient methodological detail were included, limited to human participants, specifically healthy adolescents and adults. Only English-language studies were included to maintain consistency in interpretation and data extraction. Exclusions applied to studies lacking a specific campaign focus, omitting process evaluation, or presenting solely theoretical, procedural, or methodological models without empirical application to real-world public health campaigns. Lastly, research conducted exclusively on paediatric populations or clinical groups (*e.g.* individuals undergoing medical treatment) was not considered for this review.

### Study selection and data extraction

**Figure 1:**
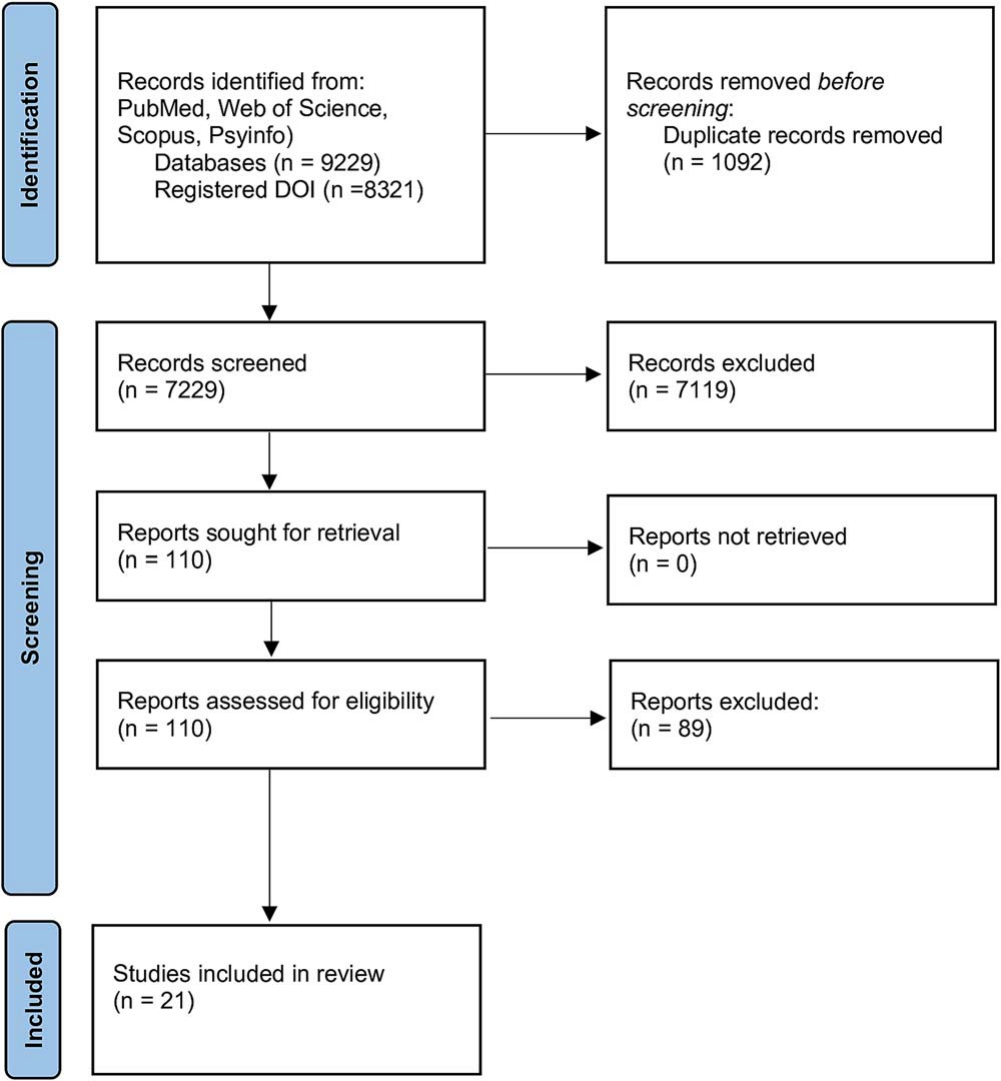
PRISMA diagram revision.

The initial search identified 9229 records. After the removal of 1092 duplicates, two reviewers independently screened the titles and abstracts of the remaining 8137 records. Of these, 7229 were excluded based on irrelevance to the research question. The full texts of the 110 potentially eligible studies were then retrieved and assessed for eligibility by two independent reviewers (TZ and GS). Following the application of predefined inclusion and exclusion criteria, 21 studies were selected for final inclusion. Any discrepancies during the screening or eligibility assessment were resolved through discussion, or by consulting a third reviewer (VB) when consensus could not be reached. The study selection process is illustrated in the PRISMA flow diagram (Fig. 1). A standardized data extraction form was developed to systematically collect relevant information from each included study. Extracted variables included: year of publication, target population, study setting, campaign type, behavioural focus (*e.g.* substance use, physical activity), methodological design, application of theoretical frameworks, components of process evaluation (*e.g.* fidelity, reach, exposure), and principal findings. Data extraction and coding were conducted independently by two reviewers. Any discrepancies were resolved through discussion and consensus to ensure accuracy and consistency. Rather than simply counting distributed materials or media spots aired, more transparent approaches included: systematic media monitoring with reach calculations [24], structured observation protocols with fidelity checklists [25], and triangulated methods combining implementation logs, stakeholder interviews, and participant surveys [26].

### Use of process evaluation findings

Process evaluation components varied substantially: 15 studies (71%) assessed reach/dose delivered, 12 (57%) examined fidelity, 11 (52%) measured participant engagement/satisfaction, 8 (38%) evaluated context/barriers, and only 4 (19%) systematically assessed recruitment processes. Measurement approaches included: standardized exposure surveys (*n* = 9), implementation fidelity checklists (*n* = 5), media monitoring data (*n* = 7), and qualitative stakeholder interviews (*n* = 11). Several studies used evaluation findings to adjust implementation strategies: some revised communication channels to better engage target audiences [30, 33, 37], others modified data collection instruments based on participant feedback [29, 34, 35], and some used insights to inform strategic planning for subsequent phases, including goal selection and thematic refinement [28, 35].

### Quality assessment

The methodological quality of the included studies was rigorously assessed using the ‘Risk Of Bias In Non randomized Studies of Interventions’ (ROBINS-I) tools, as outlined by Sterne *et al.* [27] and the revised Cochrane Risk of Bias tool for randomized trials (RoB 2), developed by Sterne *et al.* [28], was applied to assess the study's internal validity. These tools are widely recognized for their methodological robustness and comprehensive framework for evaluating the risk of bias across a range of study designs, including quantitative, qualitative, and mixed-methods research.

### Bibliometric analysis

From the 21 studies included in the systematic review, we performed a one-step forward citation analysis to expand the dataset and investigate the broader scholarly influence of these works. The resulting dataset included 373 documents published between January 1992 and March 2025, which included 314 research articles, 51 reviews, 3 conference papers, 4 editorials, and 1 note, totaling 20 288 references. We analysed this corpus using the bibliometrix package in R [29] to map influential countries, journals, authors, and keyword co-occurrence patterns.

## Results

Of the 21 studies included in this review, 13 focused on physical activity, 5 on substance use, and only 3 on public health. Details on study design, participants, assessment tools, and results are presented in the Table 1.

**Table 1:** Analysis of study characteristics and outcomes.

Study	Study design	Participants and country	Assessment tool	Results
Yeh *et al.*, 2022 [41]	Physical Activity Quasi-experimental study	*n* = 5686, United States-Mexico	TF. RE-AIM framework. PE. Reach: campaign exposure assessment across border population; dose: frequency and duration of exposure measurement. MM. Pre and post-campaign evaluations; two-item Dietary Questionnaire; physical activity measured in MET minutes.	Significant increases in fruit and vegetable consumption and physical activity, especially in the high-exposure group.
Booth *et al.*, 1992 [42]	Physical Activity Quasi-experimental study	*n* = 2426 (pre-campaign), *n* = 2474 (post-campaign), Australia	TF. Not explicitly stated. PE. Reach: national campaign coverage; awareness: message recall assessment; engagement: beliefs about physical activity. MM. National population surveys (pre and post); Risk Factor Prevalence Surveys (1983, 1989); self-reported activity/inactivity measures.	Significant increase in message recall and walking for exercise, with no significant change in moderate and vigorous activity.
Schneider *et al.*, 2013 [35]	Physical Activity Quasi-experimental study	*n* = 2307, United States	TF. Not explicitly stated. PE. Fidelity: implementation quality monitoring; exposure: campaign reach assessment. MM. Semiannual interviews; direct observations; implementation logs.	Campaign exposure was associated with increased physical activity and improved nutrition, but the effect decreased over time.
Silva *et al.*, 2020 [38]	Physical Activity Quasi-experimental study	*n* = 878 (pre-campaign, 57% female), *n* = 1319 (post-campaign, 58% female), Portugal	TF. Not explicitly stated. PE. Reach: population coverage; awareness: campaign recall; engagement: psychosocial responses. MM. Online self-administered questionnaire (pre/post); socio-demographic data collection; behavioural and psychosocial measures.	Increase in campaign awareness and self-efficacy in physical activity, with positive effects on vigorous activity.
Olscamp *et al.*, 2022 [45]	Physical Activity Observational study	*n* = 5140 (529% female), United States	TF. Transtheoretical model. PE. Exposure: campaign awareness levels; comprehension: physical activity guidelines knowledge; dose: understanding of recommended activity levels. MM. Cross-sectional online questionnaire; self-efficacy assessment scales; recent behaviour change measures.	Increased awareness of Physical Activity Guidelines and higher self-efficacy in physical activity.
Heredia *et al.*, 2017 [39]	Physical Activity Quasi-experimental study	*n* = 799 (85% female), Texas-Mexico border	TF. Not explicitly stated. PE. Dose: MVPA frequency and duration; behavioural outcomes: sedentary behaviour patterns. MM. International Physical Activity Questionnaire (IPAQ); weekly activity tracking.	Increased likelihood of meeting guidelines for moderate-vigorous physical activity and reduced sedentary behaviour in the exposed group.
Mettler *et al.*, 2000 [40]	Physical Activity Longitudinal observational study	*n* = 690 (64% female), United States	TF. Transtheoretical model. PE. Engagement: stage progression monitoring; self-efficacy: confidence changes; decision balance: pros/cons assessment. MM. Pre and post-campaign questionnaire; follow-up assessments.	Significant progress in stages of physical activity for most participants, though some regression was noted.
Emery *et al.*, 2007 [36]	Physical Activity Observational study	*n* = 175, Virginia and Wisconsin	TF. Not explicitly stated. PE. Reach: awareness assessment; fidelity: strategy implementation monitoring; context: environmental barriers/facilitators. MM. Technical assistance documentation review; telephone interviews; three-strategy evaluation: (i) increasing awareness, (ii) environmental audits, and (iii) community action.	The campaign successfully raised awareness of environmental barriers to physical activity.
Renger *et al.*, 2002 [43]	Physical Activity Quasi-experimental study	*n* = 703 pre-campaign surveys, *n* = 644 follow-up surveys, United States	FT. Theory of Planned Behaviour. PE. Baseline: pre-campaign activity assessment; post-intervention: behaviour change measurement. MM. Pre-campaign focus groups; telephone surveys; written surveys.	Decrease in the number of participants reporting no leisure-time physical activity over time.
Bélanger-Gravel *et al.*, 2017 [37]	Physical Activity Repeated measures post-test study	*n* = 692 in 2012, *n* = 676 in 2013, *n* = 686 in 2014, Canada	TF. Not explicitly stated. PE. Awareness: campaign recognition; engagement: belief changes; behavioural outcomes: physical activity patterns. MM. Focus groups; pre-test questionnaires; Youth Media Survey; School Health Action Planning and Evaluation System (SHAPE).	Modest impact on beliefs and behaviours related to physical activity.
Bélanger-Gravel *et al.*, 2014 [34]	Physical Activity Repeated measures post-test study	*n* = 803 (48% female), Canada	TF. Not explicitly stated. PE. Awareness: campaign recall; comprehension: message understanding. MM. Focus groups; web-based online surveys.	Campaign recall and recognition decreased between surveys.
Luecking *et al.*, 2021 [25]	Physical Activity Cluster randomized controlled study	*n* = 635, United State	TF. Not explicitly stated. PE. Fidelity: implementation adherence; dose: participation levels; context: barriers and facilitators. MM - Mixed-methods: surveys and semi-structured interviews; attendance logs; field notes; observation checklists.	No significant change in diet quality or physical activity minutes.
Bauman *et al.*, 2024 [24]	Physical Activity Quasi-experimental study	*n* = 1600 Australia	TF. Flowproof model. PE. Awareness: campaign recognition; comprehension: message understanding; engagement: motivation changes; behavioural outcomes: intention and behaviour changes. MM. Population survey; recognition and understanding assessment; motivation and intention measures.	Good reach, and population changes in intentions and walking behaviour.
Keijsers *et al.*, 2008 [31]	Substances Observational study	92 centers, Netherlands	TF. Not explicitly stated. PE. Context: stakeholder perspectives; fidelity: partnership effectiveness. MM. Interviews (users and operators); operator feedback collection; Drug Testing Center Monitoring (DTCM).	Improvement in collaboration between the actors involved in the campaign.
Thienpondt *et al.*, 2024 [26]	Substances Mixed-methods study	*n* = 16 547 post-campaign questionnaire (63% female), Belgium	TF. RE-AIM framework. PE. Reach: participation rates; effectiveness: health and consumption outcomes; awareness: campaign recognition. MM. Focus groups; semi-structured interviews; re/post online surveys; general health measures; alcohol consumption assessment.	High success rate in alcohol abstinence during the campaign, with physical and mental benefits.
Hong *et al.*, 2008 [32]	Substances Observational study	*n* = 4765 (51% female), United States	TF. Social Cognitive Theory. PE. Exposure: campaign reach; awareness: message recognition; engagement: emotional reactions. MM. Self-reported pre/post surveys; interviews; focus groups.	High campaign awareness among students and a positive impact on smoking-related behaviours.
Hafstad & Aarø, 1997 [51]	Substances Controlled experimental study	*n* = 3205, Norway	TF. Not explicitly stated. PE. Awareness: campaign recall; engagement: emotional reactions and theme discussions; behavioural intentions: smoking-related intentions. MM. Focus groups; pre and post-campaign surveys.	Higher campaign recall among non-smokers and different emotional reactions between men and women.
Su *et al.*, 2020 [44]	Substances Quasi-experimental study	*n* = 445 (74% female), United States	TF. Health Belief Model. PE. Beliefs: behavioural and normative beliefs; emotions: affective responses; control: perceived behavioural control; behavioural outcomes: Nonmedical use of prescription stimulants (NMUPS) consumption patterns. MM. Online pre/post surveys¡; behavioural beliefs assessment; consumption data monitoring.	Significant change in beliefs about non-medical prescription drug use and reduction in non-prescribed stimulant use.
Anwar-McHenry *et al.*, 2016 [30]	Public Health Observational study	*n* = 692 (T1), *n* = 676 (T2), *n* = 686 (T3), Australia	TF. Health Promoting Schools model. PE. Fidelity: implementation adherence; context: school-level facilitators and barriers. MM. Semiannual activity reports; semi-structured interviews with school contacts.	Greater openness towards mental health and reduction in stigma in schools involved in the campaign.
O’Hara *et al.*, 2011 [33]	Public Health Observational study	*n* = 3664 (Phone contacts), *n* = 17 098 (Website visits)	TF. Not explicitly stated. PE. Reach: contact volume (calls and website visits); dose: TV advertising exposure (TARPs); awareness: information source identification. MM. Call and website monitoring; TV advertising analysis (Target Audience Rating Points); source identification questionnaires.	Positive correlation between campaign exposure and increased contacts with GHS, with a stronger impact from 30-second ads.
Anwar-McHenry *et al.*, 2012 [30]	Public Health Cross-sectional observational study	*n* = 1113, Australia	TF. Not explicitly stated. PE. Reach: campaign coverage assessment; behavioural impact: activity changes; social impact: perceived community effects. MM. Computer-Assisted Telephone Interviews (CATI); coverage measurement; impact assessment.	The campaign reached 75% of the population, with a 20% behavioural change in exposed individuals and a reduction in stigma towards mental illness.

Note: TF: Theoretical Framework; PE: Process Evaluation; MM: Measurement Methods.

This review included 21 studies, the majority of which (*n* = 13; 62%) explicitly detailed the procedures and methods used for process evaluation. These included both structured evaluation plans and *ad hoc* implementation tracking. Nine studies (43%) employed quantitative methods, such as surveys, implementation metrics, and media monitoring [25, 26, 30–36] while eleven (52%) used qualitative approaches, such as focus groups, interviews, or content analyses, either alone or in combination, thus qualifying as mixed-methods evaluations [25, 30, 31]. Although some articles provided detailed accounts of data sources and analysis procedures, many lacked transparency regarding how process data were collected, particularly with respect to media tracking, material distribution, or stakeholder involvement. In several cases, simple counts of campaign activities were reported without explanation of data collection strategies. Only four studies (19%) clearly articulated process evaluation objectives, and even among these, the level of detail varied. Some stated campaign-specific goals (*e.g.* evaluating fidelity of implementation), while others used generic aims such as ‘to determine whether initiatives were carried out as planned.’

**Figure 2:**
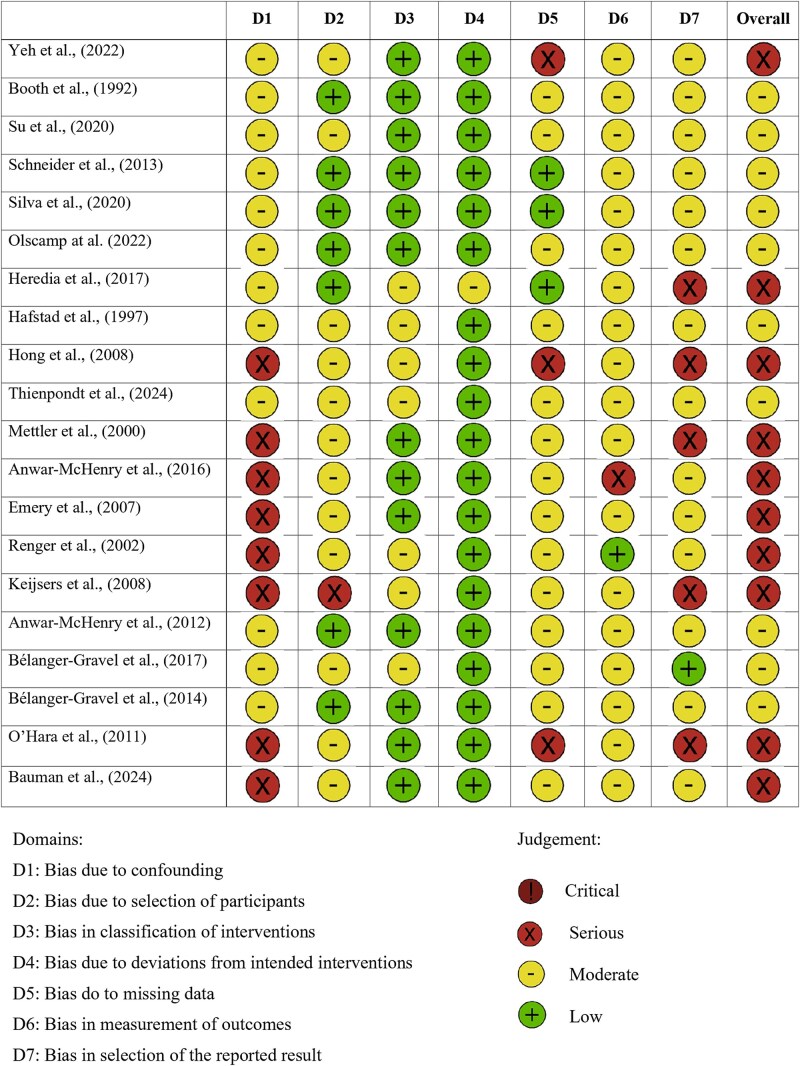
Risk of bias included in non-randomized studies.

Among the included studies, 8 (38%) explicitly described how process evaluation results were used to inform ongoing or future campaign strategies [24, 26, 30, 31, 35–38]. The reporting detail varied widely. Several studies used findings to adjust implementation methods, such as integrating new communication channels (*e.g.* interactive platforms or apps) to better engage specific audiences [26, 34, 38]. Others revised data collection instruments or evaluation tools based on participant feedback and observed limitations [31, 35, 36]. Conversely, some campaigns opted to maintain effective components identified through evaluation, such as event formats or communication tactics [25, 37]. A minority of studies [39, 40] mentioned the potential future use of results without detailing how insights would be applied. Only one study [41] quantitatively demonstrated links between process indicators, such as media exposure, and outcome variables like knowledge, attitudes, and self-efficacy. Among quantitative methods, 8 studies [24, 35, 38, 39, 41–44] (38%) employed quasi-experimental designs, 1 study [25] (5%) was a cluster-randomized controlled trial, 6 [30–33, 36, 45] (29%) used observational cross-sectional designs, and 3 [34, 37, 40] (14%) employed longitudinal cohort approaches. This distribution explains the predominance of non-randomized study designs in our risk of bias assessment (Fig. 2).

### Use of theory and framework

The integration of behavioural theory and evaluation frameworks varied considerably across studies (Table 1). Fourteen studies (67%) referenced at least one theoretical model to inform the design or evaluation of the campaign. Commonly used behaviour change theories included the Transtheoretical Model [40, 45], the Theory of Planned Behaviour [43], the Social Cognitive Theory [32], the Health Belief Model [44] and Flowproof model [24]. Some studies also employed hybrid or participatory approaches (*e.g.* Delphi techniques, psychological reactance theory). Only four studies (19%) described the use of formal evaluation frameworks, such as RE-AIM [26, 41] or the Health Promoting Schools model [30]. In contrast, five studies (23%) mentioned theoretical underpinnings without clearly identifying the frameworks used [33, 35]. Regarding formative research, only four articles (19%) provided a description of how formative activities informed campaign development. Two additional studies reported that formative research had been conducted but was published separately [39, 41].

### Reported outcomes and process evaluation integration

Physical activity campaigns (*n* = 13) predominantly reported behavioural changes including increased activity levels (62% of physical activity studies) and improved self-efficacy (46%). Substance use campaigns (*n* = 5) more frequently reported attitudinal changes including reduced stigma (60% of substance studies) and improved risk perception (40%). Public health campaigns (*n* = 3) emphasized reach metrics and awareness indicators. However, only one study [41] explicitly linked process indicators (exposure dose) to outcome magnitude, demonstrating dose–response relationships between campaign exposure and behaviour change (see Table 1).

### Risk of bias

In the evaluation of the sole cluster-randomized controlled trial conducted by Luecking *et al.* [25] the revised Cochrane Risk of Bias tool for randomized trials (RoB 2), developed by Sterne *et al.* [28], was applied to assess the study's internal validity. The assessment identified some concerns regarding the randomization process, stemming from insufficient details on sequence generation and allocation concealment. A high risk of bias was found for deviations from intended interventions, largely due to low implementation fidelity, likely affecting estimated effects. Missing outcome data posed low bias risk given adequate completeness. Concerns arose in outcome measurement due to unblinded self-reports, while selective reporting risk was low, with outcomes consistent with the prespecified protocol. Overall, the trial was judged at high risk of bias, driven predominantly by intervention deviations and limited fidelity. For the 20 non-randomized studies, risk of bias assessment using ROBINS-I (Fig. 2) revealed confounding as the most serious threat to internal validity, with 8 studies (40%) rated as serious or critical risk due to inadequate control for sociodemographic factors or baseline group differences. Selection issues represented the second major concern (30%, *n* = 6), typically stemming from convenience sampling or self-selected participation. Inconsistent exposure measurement resulted in moderate concerns for intervention classification across 12 studies (60%). Notably, outcome measurement quality was generally robust, with only 15% (*n* = 3) showing serious concerns—suggesting that weak implementation evaluation did not necessarily compromise outcome assessment. Two reviewers independently appraised study quality, resolving discrepancies through discussion or third-reviewer consultation to ensure objectivity and reliability.

### Bibliometric results

**Figure 3:**
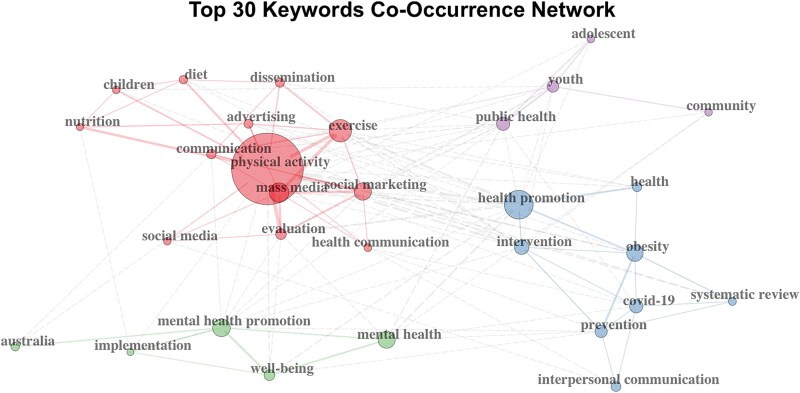
Keyword co-occurrence network. Note: Each node represents a keyword, with its size proportional to its degree (*i.e.* number of connections). Solid lines indicate co-occurrences within the same cluster, while dashed lines represent co-occurrences between different clusters. The thickness of each link reflects the frequency of co-occurrence. The colours reflect the network’s subclusters, grouping nodes based on the strength of their connections.

The bibliometric analysis identified the most highly cited documents within the extended citation network. The top three were Kahn *et al.*, (2002; 1636citations [46]), Heath (2012; 932 citations [47]), and Dobbins *et al.*, (2009; 766 citations [48]). In terms of geographic distribution, the studies citing the seed documents as well as he seed documents originated primarily from the United States (*n* = 96; Single Country Publications [SCP] = 83; Multiple Country Publications [MCP] = 13), Australia (n = 96; SCP = 84; MCP = 12), and Canada (*n* = 43; SCP = 33; MCP = 10). The journals most influenced by the seed documents were the ‘Journal of Health Communication’ (*n* = 23), ‘Health Communication’ (*n* = 16), and the ‘Health Promotion Journal of Australia’ (*n* = 11), and the ‘International Journal of Behavioural Nutrition and Physical Health’ (*n* = 11), indicating a strong presence in the field of health communication and promotion. Across the full corpus, the most frequent keywords were: physical activity (*n* = 77), health promotion (*n* = 27), mass media (*n* = 25), exercise (*n* = 24) mental health promotion (*n* = 20), social marketing (*n* = 19), evaluation (*n* = 13), evaluation (*n* = 12), mental health (*n* = 12), and intervention (*n* = 12). The main co-occurrence patterns among these keywords are visualized in Fig. 3. Notably, substance-use–related keywords (*e.g.* ‘drug use,’ ‘stimulants,’ ‘doping’) appeared infrequently or not at all in the network, which is meaningful given the centrality of substance use in our review and suggests a gap in the visibility and dissemination of substance-use campaign research.

## Discussion

This systematic review highlights that while many public health campaigns on physical activity, substance use, and body image include process evaluations, there is considerable inconsistency in how these are conducted and reported. Although physical activity benefits are well-established, campaigns promoting physical activity may inadvertently face challenges when target populations simultaneously engage in risky behaviours such as performance-enhancing drug use, creating a paradox where health-promoting behaviours coexist with substance misuse. Among the 21 reviewed studies, 62% reported some form of process evaluation, mostly using quantitative measures such as media exposure and message recall. However, only 19% of studies clearly articulated the objectives of their process evaluations. Detailed assessment of implementation fidelity and stakeholder involvement was often missing. These findings emphasize the need for standardized, transparent process evaluation frameworks to improve the design and effectiveness of future campaigns [18, 21].

Inconsistent use of theory further limited the interpretability and comparability of findings. Although over half the studies referenced behavioural theories, including the Transtheoretical Model, Social Cognitive Theory, and the Theory of Planned Behaviour [32, 40, 43], few applied these models systematically to guide evaluation strategies. Similarly, structured frameworks such as RE-AIM or the CDC Evaluation Framework [19] were rarely employed [26, 41], and formative research was seldom integrated into campaign development. These limitations may constrain both the replicability and scalability of interventions across different populations and settings.

Bias due to confounding emerged as the most serious issue, followed by bias in the selection of reported results. This matters because confounding reflects structural weaknesses in study design and limits confidence in attributing changes to the campaign itself. Had the highest risk been found in deviations from intended interventions or other domains, concerns would instead relate to implementation failures. As shown in Fig. 2, the pattern we observed highlights design- and analysis-related vulnerabilities that remain largely unaddressed in previous discussions.

Critically, only one study [41] quantitatively linked process indicators to outcome variables, highlighting a common disconnect between implementation assessment and evaluation of effectiveness. This gap limits the ability to understand how and why campaigns succeed or fail, weakening the evidence base for health communication strategies.

Although our search strategy equally targeted both behavioural domains, substance use campaigns comprised only 24% of our sample, compared to 62% focusing on physical activity. This striking disparity suggests that rigorous process evaluation has been adopted far less systematically in substance use prevention, a concerning gap given the public health urgency of addressing drug-related harms.

A minority of studies demonstrated how process evaluation findings informed practical adjustments to campaign design or delivery [31, 35, 36, 38]. These included the integration of new communication channels, modifications to evaluation tools, and strategic planning for subsequent phases. However, such examples remain exceptions rather than the norm.

The bibliometric analysis shows research is concentrated in few countries and journals, with dominant themes like physical activity, mass media, and health promotion reflecting the field’s interdisciplinary scope across public health, psychology, and communication.

Our findings align with previous reviews documenting persistent methodological gaps. Getachew-Smith *et al.* [49] reported that only 34% of health communication campaigns clearly defined process evaluation objectives; we found an even lower rate (19%), suggesting limited methodological progress. Similarly, Johansson *et al.* [16] noted that interventions frequently measure reach and dose but neglect fidelity and context, a pattern we confirmed (71% assessed reach versus 57% fidelity, 38% context). The theoretical gap we identified also reflects Lim *et al.*'s (2023) [23] finding that merely 28% of health promotion interventions employed formal frameworks like RE-AIM; our review found only 19% used such structured approaches. Moreover, few evaluations are grounded in theoretical or conceptual frameworks, and even fewer report using validated instruments to support structured analysis [50]. Most critically, consistent with Getachew-Smith *et al.* [48], we found that process evaluations remain predominantly descriptive: only one study (5%) quantitatively linked process indicators to outcomes, representing no improvement over two decades and confirming the persistent gap between recognizing process evaluation's importance and implementing it rigorously [41].

From a practical standpoint, there is a clear need to enhance the methodological rigour and utility of process evaluations. Future research should prioritize the integration of theory-driven frameworks, clearly defined evaluation goals, and mixed-methods approaches capable of linking implementation quality to outcomes. Adopting validated tools and consistent reporting standards will help advance the field and support more actionable, comparable findings.

Ultimately, this review underscores a persistent challenge: although process evaluation is recognized as essential for understanding public health campaign effectiveness, its application remains inconsistent and underdeveloped. Overcoming this gap necessitates strengthened methodological frameworks and a cultural shift towards embedding evaluation as an integral component of campaign design. Prioritizing capacity-building for continuous, theory-informed evaluation, and ensuring systematic use of findings to refine and scale interventions, is imperative for enhancing the impact, sustainability, and accountability of future health communication initiatives.

### Strengths

This review provides several important contributions. It represents the first systematic synthesis of process evaluation practices across both substance use and physical activity campaigns, revealing differential methodological approaches between these interconnected domains. Rigorous PRISMA 2020 methodology was employed with dual independent screening, comprehensive searches across six databases plus grey literature, and validated quality assessment using RoB 2 and ROBINS-I tools appropriate for diverse study designs.

The integration of bibliometric analysis adds unique value, contextualizing findings within broader scholarly trends across 345 publications and three decades. Critically, our explicit focus on evaluation methodology, rather than campaign effectiveness, addresses an underdeveloped area, systematically documenting which process components are assessed, which frameworks guide evaluation, and crucially, identifying that only 5% of studies link process indicators to outcomes. This gap analysis provides concrete guidance for strengthening future campaign evaluation.

### Limitations

The findings of this review are constrained by the inclusion of English-language studies only, which may have excluded non-Western perspectives, and by considerable heterogeneity in study design, populations, objectives, and reporting, limiting cross-study comparability, precluding meta-analysis, and restricting structured synthesis of process evaluation components. Another limitation relates to the poor methodological transparency in many studies, with insufficient details on data collection, theory, and evaluation. Despite using RoB 2 and ROBINS-I, assessing bias in diverse, non-randomized real-world interventions remains inherently challenging and complex [27, 28].

Lastly, despite protocol registration and dual screening, data extraction lacked double-blind coding and inter-rater reliability testing, potentially affecting classification consistency. Moreover, while key process dimensions were identified, their contributions to campaign outcomes were not assessed, warranting future meta-evaluative investigation.

## Conclusions

This review highlights the pivotal role of process and implementation evaluations in enhancing the transparency, effectiveness, and adaptability of public health campaigns focused on physical activity and substance use. While many of these initiatives yield positive outcomes, their overall impact is often limited by inconsistent evaluation methods, limited use of formal frameworks, and insufficient integration of process data to guide improvements.

To overcome these challenges and advance the field, future evaluations should prioritize: i) clear articulation of process-related objectives; ii) consistent application of theoretical and evaluative models (*e.g.* RE-AIM, CDC guidelines); iii) integration of mixed-methods approaches to gain deeper insight; iv) transparent documentation of implementation procedures; and v) systematic use of findings to refine campaign strategies and support scalability.

Mixed-methods approaches provide critical depth to process evaluation by combining quantitative tracking of core indicators (reach, exposure, fidelity) with qualitative exploration of context and mechanisms. As emphasized in RE-AIM and CDC frameworks, this integration enables evaluators to determine not only whether campaigns achieved implementation targets, but why they succeeded or failed insights essential for replication and adaptation across settings.

Far from being a mere methodological exercise, robust process evaluation is not merely methodological but a strategic means to improve quality, accountability, and sustainability of public health interventions. In complex digital contexts, embedding theory-driven, participatory, well-documented approaches is vital. This review synthesizes current practices, identifies gaps, and offers guidance. Despite recognition, process evaluations remain inconsistently applied, theoretically disconnected, and underdeveloped; strengthening them through validated tools, coherent frameworks, and structured feedback can enhance campaign effectiveness.

## Data Availability

The data underlying this article will be shared on reasonable request to the corresponding author. The review protocol was pre-registered on PROSPERO (CRD420251054292).
